# Supplementary material for: Patterns of Intron Gain and Loss in Fungi
Source: PLoS Biol. 2004 Nov 30;2(12):e422. doi: 10.1371/journal.pbio.0020422 (PMC532390; doi:10.1371/journal.pbio.0020422)
Supplement: Table S1 — Also available at http://genes.mit.edu/NielsenEtAl/. (4.3 MB ZIP). [file pbio.0020422.st001.zip › NielsenEtAl/html/169.html]

AN0561.1.NCU05226.1.MG04146.1.FG09732.1


```
 CLUSTAL W (1.82) Multiple Sequence Alignments - Introns Inserted


Sequence 1: NCU05226.1	694 aa
Sequence 2: FG09732.1	654 aa
Sequence 3: MG04146.1	683 aa
Sequence 4: AN0561.1	756 aa
Alignment Length: 790 aa
Number Identitical Residues: 236 aa
Alignment Score (without introns) 12583


MG04146.1 	--------------------MRALY--SSVRAP--ILRISRGTFYRHHPSAQVS---HPN
NCU05226.1	--------------------MRFPPPGTTVRPP--IIRITNGTFYRHQPSSHGHGHGHPN
FG09732.1 	--------------------MRP----LTSCPP--IIRIANGTFYRHHPNTTTT-SLRPN
AN0561.1  	MLRLPPLRTRLVRPCSVPSVVRALSSSSSSSPPPPLVRIENATFYENYPTPEDE-AKGAN
          	    ..  :    ..: .: :*   .. :  .*..::** ..***.: *..       .*

MG04146.1 	PPLFKDLDFELSSTAQLANGEKQQHWCIVGPSLSGKTTFLEVLRGFHLSIPPTARSYPYL
NCU05226.1	PPLFSNLTLDIPSHPSSP-----HNWAILGPSQSGKTSFLQLLRGQYLCFPPTARSFPYL
FG09732.1 	PPLFSDLNFELPSTSNSP-----RNWCIVGPSLSGKTTFLQALKGRYLCDPPIARSYPYL
AN0561.1  	PPLFPNLNFLLPSQPGKEG---HQHWAIIGSP--CRTQFLNVLRGQYICIPPTGRSYPYL
          	**** :* : :.* .   .    ::*.*:*..   :* **: *:* ::. ** .**:***

MG04146.1 	STDAFP---CHLR-TPHQAIQYVGFDASSSS-GLG--PPTSTYLAARYESRREDTDFSLR
NCU05226.1	STEDVP---TRLRGNPAKAIQYVGFDATSTSGGLG--AAASSYISARYESHREQTDFSVK
FG09732.1 	SSENVP---KRLR-TPQKAIQYVGFDAEQTGGGLGSSVATSAYLSARYESRREITDFSLR
AN0561.1  	LTDNIAQKDTRLR-DPANAIQYIGFSGEGSG-AIG--GTRGAYLSARYESLREETDWTVQ
          	 :: ..... :**  * :****:**..  :. .:*   . .:*::***** ** **::::

MG04146.1 	DYLLGNTELNPAEKPG--QSAAEEEKLLGRVVHDLKLAPLLELPVSFLSNGQGCRARIAR
NCU05226.1	DWLLGNTELNPTSMPG---DYKVDEKLFERVVVDLRLDTLLDLPVSFLSNGQGRRARIAR
FG09732.1 	DFLLGNTELNPMKTEQGDDDASIPLGLLSRVIKDLKLENLLDLPVSFLSNGQGRRARIAR
AN0561.1  	QYLKGQTSLNPLEEEK--DGTVRDEDLFFRVVKDLRLGDLLDMPVANLSNGQTRRTRIAK
          	::* *:*.*** .     ..      *: **: **:*  **::**: *****  *:***:

MG04146.1 	ALLRTPEVLLLDEPFM~GLDPPTVVALSPFLRGLAEQAAPRLVLSIRPQDPLPSWISHIV
NCU05226.1	ALFKDPEVLLLDEPFM~GLDPATVAGLSPLLQSLAEKKSPRLVLAARPQDPLPEWITHLV
FG09732.1 	ALLTKPEVLLLDEPFM~GLDPPTVSGLSPLLYSLSEKASPRLVLSARPQDPVPDWITHLV
AN0561.1  	ALLGKPELLLLDDPFM1GLDPATVRSISELLHRLANKSDPRLVLSLRPQDPIPDWITHLL
          	**:  **:****:*** ****.** .:* :*  *:::  *****: *****:*.**:*::

MG04146.1 	YLGNNCQVASIGQKEDVMDQLRSYVSRVADSK--TPEDPTLPVSALREMGRTLTDDG-VE
NCU05226.1	YLRTDSQVGAMGERGTVLDGLRAYVRGVWKGG--LSEDETMPVHALIDIGRTLTKEG-IK
FG09732.1 	YLRTDCQVGSMGPKEEVLEGLKKYVHGVRKGG--LVEDEKLPLHSLVDIGRVLPSAASTE
AN0561.1  	VTGHSNRVLLQATRAEAQKHFVVWAKKTGNSRRINANERAIAAKVQGDIQAGYLDKQLVF
          	    . :*   . :  . . :  :.  . ..     ::  :.     ::     .     

MG04146.1 	GVVFDAVGLQSSIAMPELP-----RAAINGATSIGEPLVEMNGVQVGYGDKIVLGNWDND
NCU05226.1	GEGLAEALTRSPTNQAPIIDSAPGPEKTAPEEAEKEPLVEMSGVTVRYGSKTVLGNWP--
FG09732.1 	GSGHTEQSS---------------FKELEPSKPGAEPLVEMDGCRVQYRGKVALGNWAQQ
AN0561.1  	DLQLVPSSEE--------------NPVQFQMPPDGEALIEMGGVRVQYGDKVVLGGWSQN
          	.                               .  *.*:**.*  * * .* .**.* ..

MG04146.1 	TITGQTGQKGLVWTVRRGERWGIFGANGSGK0TTLFSLLGSDHPQCYSQPVKLFGRSRLP
NCU05226.1	--------SGLHWTVRRGSRWGVFGPNGSGK~TTIVSLLCSDHPQTYSLPIKLFGRSRLP
FG09732.1 	TADGSK--DGLIWTVRRGERWGVFGPNGSGK~TTIVSLLCSDHPQSYSLPIKLFGRSRLP
AN0561.1  	IRKKPQ--EGLHWTVRRGQRWVILGPNGCGK~TTLLSLITSDHPQTYAQPIKLFGRSRLP
          	        .** ******.** ::*.**.** **:.**: ***** *: *:*********

MG04146.1 	EPGSGQPPLTFWDIQERIGHSSPEIHGAIPRRLSVRRVLETAWAETFISTPKLEKNASEK
NCU05226.1	EPGSGQRPLTFWDIQSRVGHSSPEIHQYMPRRLTVRSVLESAWADTFSSVPKLTPEATGK
FG09732.1 	EPGSSQRPLTFWDIQSRIGHSSPEVHQHMPRSLTIRQTIENAWSETFRAKPKLNDAAKEK
AN0561.1  	EPG--KPPISLFELQSRIGHSSPEIHAFFPRQLTVRQAIESAFAETFLSKPALDHDRDLD
          	***  : *::::::*.*:******:*  :** *::* .:*.*:::** : * *      .

MG04146.1 	VEATLVWFERELNPAVRREN--------------EQTN------HGSGSSTLKWADDYLF
NCU05226.1	VDATLSWFAHELNPSFAKRS--------------HHTPVELAETDADDEASLKWAKDYQF
FG09732.1 	VEAALRWFAPELHPAYSSSS--------------HEN--------------LSWADDYMF
AN0561.1  	VSAALRFFRPELDPNFKASLKEGPPSVWLNRQNFPETSKTFKGRTFEPEYDVEYADTLTF
          	*.*:* :*  **.*      .....:   . ..  ..         . .  :.:*.   *

MG04146.1 	GELSFSAQRVLLLLRAIIKSPDIVVLDEAFSGMDEAVRDKCMLFIAHGEEKILAARGGRQ
NCU05226.1	GELPFSSQRLLLFLRAIVKNPDIVVLDEAFSGMDDAVRDKCMLFLMHGENKTFASSSSSS
FG09732.1 	GGLSFSAQRIALFIRSMIKSPDVVVLDEAFSGMDDAVRDKCTLFLEQGESRTYRGE----
AN0561.1  	GQLSTPQQRLVLFLRALIHKPDIVILDEPFSGMSSSVRDKCIHFLEVGERNAVSTATRRA
          	* *. . **: *::*::::.**:*:***.****..:*****  *:  ** .         

MG04146.1 	--------------------VMVDSQAKQDGNVRVTGLSDRQAFICISHVKEEVPDFVRE
NCU05226.1	SSSTSAPTTKTKNGLSVAPVEVVDSEQAKAGKVKVHGLTDEQALICISHIKEEVPDCVRE
FG09732.1 	--------------------DIVESDASKSGQVKITGLSDEQALICIAHVKEEVPDCVRE
AN0561.1  	GGKNPWTVAVTGD----EGDKDVRFQRNILAEKRHFGLTDQQALIMISHLREEIPDFVRH
          	..... . : : .         *  :    .: :  **:*.**:* *:*::**:** **.

MG04146.1 	WLCLPEAT----SRLPARFGRLDG----------PLRLDGKRWDD~IWGMGRGAR2KGLE
NCU05226.1	WVCLPEAS----SGQEARFGRLDG----------PLRLSGRRWGE~VWGVNGN--~----
FG09732.1 	WLCLPEAN----TGSPARFGRLDG----------PLRTDEKRWHE0HTVKLKVIH~VME-
AN0561.1  	YIRLPSAQNDDATGLDFRFGYLKGKNALRQPPVWDLAWTHKDKFE~SMGARRNFR~RAPD
          	:: **.* ...::    *** *.*..:  ...   *    :   :              .

MG04146.1 	NGQLF---------
NCU05226.1	--------------
FG09732.1 	--------------
AN0561.1  	SNETDEDVYEYWSI
          	...  ..  .  :
```
